# Ability of 2 estimation methods of body fat percentage in identifying unfavorable levels of cardiometabolic biomarkers in adolescents: Results from the LabMed study

**DOI:** 10.1097/j.pbj.0000000000000052

**Published:** 2019-09-04

**Authors:** José Oliveira-Santos, Jorge Mota, Carla Moreira, Sandra Abreu, Luís Lopes, César Agostinis-Sobrinho, Rute Santos

**Affiliations:** a Faculty of Sport, Research Centre in Physical Activity, Health and Leisure (CIAFEL), University of Porto, Portugal.; b Faculty of Social Sciences, Early Start Research Institute, University of Wollongong, Wollongong, NSW, Australia.

**Keywords:** Adolescents, body fat, cardiometabolic biomarkers, ROC curves

## Abstract

**Background:**

To assess and compare the ability of body fat percentage (BF%) estimated by 2 methods, bioelectrical impedance analysis (BIA) and by the Slaughter et al equations for triceps and subscapular skinfold thickness (SKF), in identifying unfavorable levels of several biomarkers of cardiometabolic risk.

**Methods:**

Cross-sectional school-based study with 529 apparently healthy adolescents (267 girls), aged 14.3 ± 1.7 years.

**Results:**

BF% estimated by both methods always showed higher areas under the curve (AUC) for each biomarker in girls than in boys (with the exception of BIA for leptin). BF% estimated by BIA and by SKF presented a discriminatory ability in identifying unfavorable levels in all biomarkers of cardiometabolic risk in girls; however, BF% estimated by BIA displayed the highest AUC (except for C-reactive protein). In boys, BF% estimated by SKF presented higher AUC for C-reactive protein, fibrinogen and erythrocyte sedimentation rate; and BF% estimated by BIA for complement C3 and leptin. Positive and significant associations between BIA and SKF with all biomarkers (*P* < .05) were found (except for SKF and complement C4 in girls, and SKF and fibrinogen and erythrocyte sedimentation rate in boys), after adjustments for pubertal stage, cardiorespiratory fitness, adherence to the Mediterranean diet and socioeconomic status.

**Conclusions:**

Overall, diagnostic performance was more accurate in girls. BF% estimated by BIA presented a slightly better overall discriminatory ability for each biomarker than BF% estimated by SKF in girls, while in boys no method clearly prevailed over the other.

## Introduction

Adipose tissue is not recognized anymore simply as fat storage but is now accepted as an endocrine organ, and an important source of biologically active substances with local and/or systemic action.^1^ However, the ability of the adipose tissue to develop excessively also poses a series of molecular and cellular alterations affecting systemic metabolism,^2^ promoting a state of chronic low-grade systemic inflammation even in pediatric ages,^3^ a condition that tends to track from childhood to adolescence and from adolescence to adulthood.^4^ For example, increases in fat mass during the pubertal growth of Finnish girls^5^ were able to explain the increases in high-sensitivity C-reactive protein (CRP) levels 7.5 years later. In addition, data from the Bogalusa study^6^ showed that body fatness in childhood was the major predictor of higher CRP and lower adiponectin levels in young adulthood. The early development of excessive adiposity and its circumstances appear thereby to contribute to an increased risk and to the premature onset of a series of cardiometabolic disorders,^7,8^ and as such, its close monitoring is important.^9^

Skinfold thickness (SKF) measurements and bioelectrical impedance analysis (BIA) are 2 nonlaboratory methods frequently used to assess adiposity and to estimate body fat percentage (BF%).^10^ The method of SKF measurements is based on the idea that a collective measure of subcutaneous adipose tissue from various sites on the body may provide a good estimate of total body fat,^11,12^ and several site-specific skinfold measurements are used in regression equations (eg, of Slaughter et al^13^) to estimate BF% in children and adolescents. The methodology of SKF measurements is quite simple in its essence,^14^ and has been widely used for measurement of body fat in epidemiological settings.^10^ However, it requires well-trained assessors who can master the procedures of determining the measurement site, pinching the skin, and handling and reading the calliper to avoid errors.

Due to its simplicity (no special skill or training is needed from the operators), quickness, painless, and noninvasive characteristics, and increasingly cheaper, BIA is also considered a practical method to estimate BF% in adolescents^15^ on epidemiological settings, such as in school-based studies, and in groups of the same ethnicity and without any underlying medical conditions.^16,17^ This technique measures the impedance of the body to a small electrical current, and is based on the premise that lean tissue acts as a good electrical conductor, while fat resists the transmission of the electrical impulse. However, some inconsistencies have been reported when BIA is compared with reference laboratorial methods, highlighting the need for further analysis.^18^

Although the broadly use of these techniques in epidemiological studies, to the best of our knowledge, no study have explored and compared the ability of BF% estimations by these methods in identifying increased levels of biomarkers of cardiometabolic risk in adolescents. Therefore, this study aimed (i) to assess and compare the ability of these 2 methods in identifying unfavorable levels of several biomarkers of cardiometabolic risk in apparently healthy adolescents, for an early signaling of potential risk of cardiometabolic disorders; and (ii) to compare BF% estimations using BIA or SKF.

## Methods

### Study design and sampling

Data for this study were obtained in the fall of 2011 from the baseline data collection of the Longitudinal Analysis of Biomarkers and Environmental Determinants of Physical Activity Study (LabMed Physical Activity Study), a school-based prospective cohort study carried out in 5 schools from 4 cities in the north of Portugal (Barcelos, Braga, Vila Nova de Gaia, and Ílhavo), which aimed to evaluate the independent and combined associations of dietary intake and fitness levels on blood pressure levels of adolescents. Study design, sampling, and procedure are reported elsewhere.^19,20^ From 1229 apparently healthy adolescents (ie, without any medical diagnose of physical or mental impairment) that agreed to participate in the LabMed study, 534 accepted to undergo blood collection. This subsample did not differ in any variable from the rest of the adolescents whose blood has not been tested (data not shown). Of the 534, 5 were later excluded from this analysis due to high-sensitivity CRP values >10 mg/L, which may be indicative of acute inflammation or illness,^21^ leaving 529 adolescents (267 girls and 262 boys, mean age 14.3 ± 1.7 years) as the final sample for the present study. This number fulfills the condition of a receiver operating characteristic (ROC) sample size calculation (providing 80% power at 5% significance, for a minimum expected area under the curve [AUC] of 0.6 and null hypothesis value of 0.5), requiring at least 514 subjects for the present analysis.

This study was conducted according to the guidelines laid down in the Declaration of Helsinki and all procedures involving human subjects were approved by the Portuguese Data Protection Authority (No. 1112434/2011), the Portuguese Ministry of Science and Education (No. 0246200001/2011), and the Ethics Committee of the Faculty of Sport from the University of Porto (No. CEFADE 01.2014). Written informed consent was obtained from all participants and their parents or legal guardians.

### Assessments

#### Anthropometric measurements

All anthropometric measurements were performed in a private place, in the presence of 2 researchers of the same sex as the participant, with all adolescents lightly dressed with a t-shirt, shorts, and barefoot.

Bodyweight was measured to the nearest 0.1 kg, using a portable electronic weight scale (Tanita Inner Scan BC 532, Tokyo, Japan). Body height was measured to the nearest 0.1 cm, with the adolescent standing upright against a portable stadiometer (Seca 213, Hamburg, Germany). BMI was calculated as weight divided to height squared (kg/m^2^), and the participants were classified as underweight, normal weight, overweight, or obese using the age and sex-specific cut-off values proposed by the former International Obesity Task Force (now called World Obesity/Policy and Prevention).^22,23^

BF% was estimated by 2 different methods: BIA and SKF.

A frequency current of 50 kHz (Tanita Inner Scan BC 532) was used to measure leg-to-leg BIA. After manual introduction of the sex, age, and height into the scale system, participants were asked to come up and remain still on the scale until measurement was completed, fulfilling the manufacturer's instructions. This scale is suitable for measuring BF% in participants with an age range from 7 to 99 years old according to the instruction manual, and has already been used for the same purpose in other studies with adolescents.^24^ Given that previous research has shown inconsistent data on the effects of hydration status and recent exercise on BIA derived body composition measurements,^25,26^ we asked participants to fast overnight, not to drink any fluid 4 hours prior to their test, urinate within 30 minutes of test and not to perform any physical exercise in that morning, in order to minimize eventual errors and variations in the BF% assessment.

Triceps and subscapular SKF were measured on the nondominant side of the participant's body, with a newly calibrated skinfold calliper (Harpenden Skinfold Calliper Model HSB-BI, UK) with a constant pressure of 10 g/mm^2^, by the same individual to avoid interobserver errors. The participant stood comfortably stand, with arms hanging relaxed at the side and with shirt off. All measurements were performed according to standardized procedures^14^ as follows: subscapular skinfold was measured on a diagonal, inclined inferior-laterally approximately 45° to the horizontal plane in the natural cleavage lines of the skin (inferior to the lower angle of the scapula); triceps skinfold was measured on the mid-point of the posterior side of the arm (half the distance between the acromion and the olecranon). The SKF were pinched 1 cm above the measurement site, for 3 seconds, and the mean value of 2 nonconsecutive measurements was recorded to the nearest 0.1 mm. Slaughter et al^13^ equations considering sex and pubertal stage were used to estimate BF% derived from these SKF measurements.

#### Pubertal stage assessment

Pubertal stages (A—breast development in girls; genital development in boys; and B—pubic hair development, for both sexes) were self-assessed by the participants according to the classification by Tanner Stages,^27^ in a private place, and then communicated to a researcher of the same sex in a closed envelope, with stage 1 being prepubertal and 5 being adult.

#### Biochemical assessment

Blood samples were collected by venepuncture from the antecubital vein between 8:00 and 10:00 am after an overnight fast (>10 hours), stored in sterile blood collection tubes in refrigerated conditions (4–8°C) during the morning period (no longer than 4 hours), and then delivered to an analytical laboratory for testing according to standardized procedures, as follows: (i) high sensitivity CRP (latex enhanced immunoturbidimetric assay [Siemens ADVIA 1800, Erlangen, Germany)]; (ii) fibrinogen [Clauss assay (Siemens BCS XP System, Erlangen, Germany]); (iii) adiponectin and leptin (enzyme-linked immunosorbent assay Plate Reader); (iv) complement factor C3 (C3) and complement factor C4 (C4) (polyethylene glycol enhanced immunoturbidimetric assay [Siemens ADVIA 1800]); and (v) erythrocyte sedimentation rate (ESR) (Westergren method [Starrsed, RR Mechanotronics, The Netherlands]). CRP, C3, C4, adiponectin, and leptin were determined in serum, fibrinogen was determined in plasma and ESR was determined in whole blood. Existing literature shows that the biomarkers analyzed in this study have been increasingly explored in studies involving children or adolescents,^28–32^ suggesting that they are a valid choice for this age group.

#### Cardiorespiratory fitness

Cardiorespiratory fitness was estimated by the Léger equation^33^ as the maximal oxygen consumption (VO_2max_, mL/kg per minute), using data from the 20-m shuttle run test, which has proven to be a feasible, valid, and reliable field test in young people.^34^ Participants were required to run straight back and forth between 2 lines set 20 m apart. Running speed started at 8.5 km/h, increasing levels by 0.5 km/h each minute, and reaching 18 km/h at minute 20. Each level was announced on a sound device. Participants were instructed to keep pace according to the audio signals until exhaustion, and encouraged to keep running as long as possible throughout the course of the test. The test was finished when the participant failed to reach the end lines concurrent with the audio signals on 2 consecutive occasions, or when the subject stopped because of fatigue. The test was performed once, and the total number of shuttles performed by each participant was recorded to posterior calculation of VO_2max_ using Léger's equations.

#### Adherence to the Mediterranean diet

The KIDMED index^35^ (Mediterranean Diet Quality Index for children and adolescents) was used to assess the degree of adherence to the Mediterranean diet, considered a healthy dietary model and associated with a lower occurrence of cardiometabolic diseases^36^ and certain cancer types.^37^ This index is based on 16 questions self-administered, which sustain principles of Mediterranean dietary patterns as well as those that undermine it. Questions indicating a negative connotation with respect to the Mediterranean diet were assigned a value of −1 and those with a positive aspect +1. The sum of the values ranges from 0 to 12, where a higher index means good adherence to the Mediterranean diet.

#### Socioeconomic status

The Family Affluence Scale (FAS)^38^ was used as a proxy measure of adolescent's socioeconomic status. This scale is a 4-item questionnaire regarding information on vehicles, home, lifestyle, and access to technology, with a range of scores from 0 to 9 points, that allows adolescents to indirectly report their family income, with the highest score corresponding to the highest socioeconomic level.

### Statistical analyses

Two-sided Student *t*-test was used for comparisons between groups, for continuous variables.

A *Z*-score (*Z*-score = [participant's value − mean value of the sample]/standard deviation) was computed by age and gender for each biomarker, and an increased level was considered when the individual had ≥1 SD of the *Z*-score, except for adiponectin, where decreased level was considered when the individual had ≤−1 SD of the Z-score. In the absence of consensual age and sex reference ranges’ values for each biomarker analyzed, this procedure allowed us to identify the adolescents presenting the highest values for each biomarker (or lowest, in the case of adiponectin), and consequently create 2 categories for the ROC curves analysis.

ROC curves were used to analyze the ability of the 2 methods of BF% estimation in discriminating between low/high values of biomarkers of cardiometabolic risk, providing the best trade-off between sensitivity and specificity of each adiposity measure and respective cut-off value. The AUC, ranging between 0 and 1 (a worthless and a perfect test, respectively), is a global indicator of diagnostic performance, and represents the ability of the test to correctly classify the participants with high or low levels of biomarkers. ROC curves analysis showed how well each method performed in identifying unfavorable levels of biomarkers, as indicated by *P* values <.05 and an AUC >0.6. Cut-off points were chosen based in the highest Youden index, that is, the point on the ROC curve that is farthest from line of equality.

Based on the cut-off values suggested by the ROC curves, logistic regression analyses were performed to further study the relationships between the estimation methods of BF% and each biomarker, adjusted for the following potential confounders: pubertal stage (in the case of BF% by BIA), cardiorespiratory fitness (VO_2max_), socioeconomic status, and the degree of adherence to a Mediterranean diet (KIDMED index). Regression models were constructed only when the estimation method of BF%, in prior ROC analysis, presented discriminatory ability (AUC > 0.6 and *P* < .05) for the biomarker in question. Variables were tested simultaneously.

MedCalc statistical software version 15 (MedCalc software, Mariakerke, Belgium) was used for the ROC curves analyses. All other statistical analyses were performed using the Statistical Package for Social Sciences version 22.0 (SPSS, Inc. IBM Company, New York, NY). A *P* < .05 denoted statistical significance.

## Results

Descriptive characteristics are presented in Table 1. Boys were heavier and taller than girls, while girls presented higher values of BF% than boys in both estimation methods (*P* < .05 for all). In boys, the mean values of BF% were significantly higher when assessed by SKF (19.54%) than with BIA (15.86%), *P* < .001.

**Table 1:**
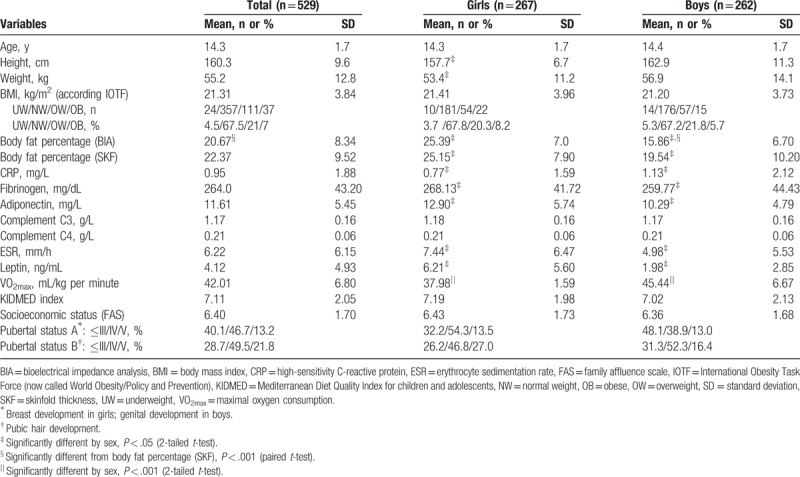
Descriptive characteristics of the study sample

CRP values were higher in boys, whereas fibrinogen, adiponectin, ESR, and leptin were higher in girls (*P* < .05 for all).

Boys presented higher levels of cardiorespiratory fitness (*P* < .01) than girls, and no differences on socioeconomic status (FAS) and the degree of adherence to a Mediterranean diet (KIDMED index) were found between sexes.

Table 2 presents the parameters of the ROC curves analyses for the diagnostic performance of each estimation method of BF% (BIA and SKF) in identifying unfavorable levels of biomarkers in girls and boys. Values in bold indicate which method presented better predictive power (greater AUC) for each biomarker.

**Table 2:**
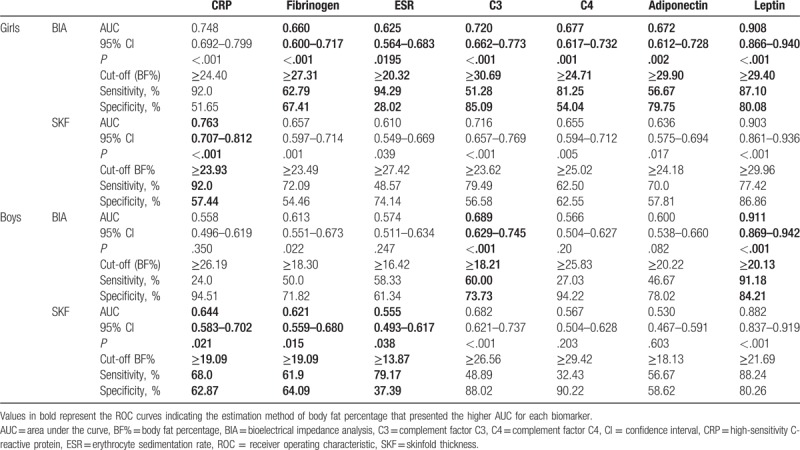
Parameters of the ROC curves analysis for the diagnostic performance of BIA and SKF, in identifying unfavorable levels of biomarkers of cardiometabolic risk in girls and boys

Both methods showed significant AUC for all the biomarkers in girls, but BIA presented a slightly better discriminatory power for all biomarkers, with the exception for CRP.

In boys, the results were more heterogeneous, with no predominance of 1 method over the other, when considering all biomarkers. SKF presented the highest AUCs for CRP, fibrinogen, and ESR, while BIA presented the highest AUCs for C3 and leptin. The lower limit of the 95% CI of SKF for ESR is very close to 0.5 (0.493–0.617), suggesting poor diagnostic performance. None of the methods presented discriminatory ability for C4 and adiponectin in boys.

As shown in Tables 3 and 4, the number (%) of adolescents below or above the cut-off values suggested by previous ROC curves analyses (see Table 2), and the respective mean values of each biomarker in question are provided. In addition, logistic regression analyses adjusted for pubertal stage, cardiorespiratory fitness, socioeconomic status, and KIDMED index were performed for the estimation methods of BF% that revealed discriminatory ability (ie, AUC > 0.5 and *P* < .05) for each biomarker. Positive and significant associations with all biomarkers (*P* < .05) were found, with the exception of SKF for C4, in girls, and also of SKF for fibrinogen and ESR in boys, after adjustments for the above-mentioned confounders.

**Table 3:**
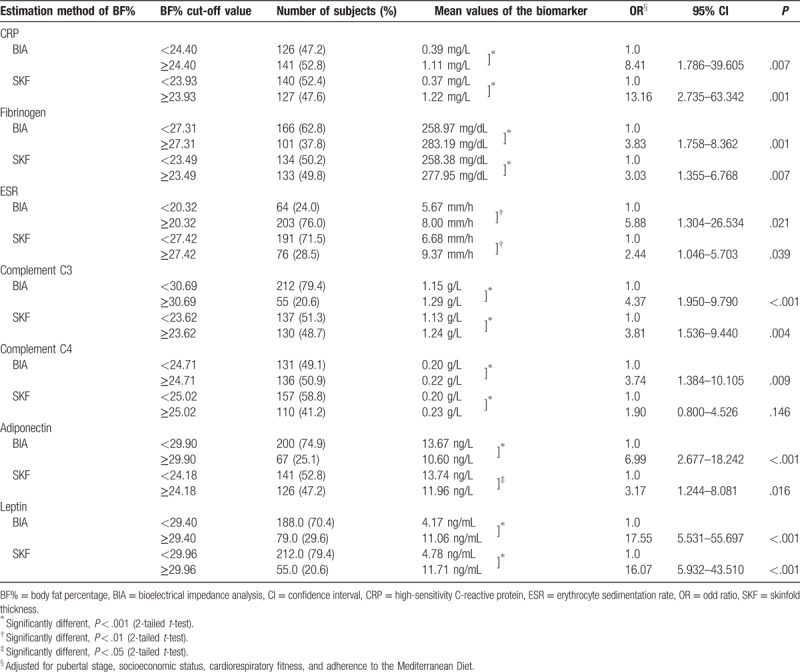
Number and percentage of girls below and above the cut-off value and respective mean values of the biomarker in question, and associations between the estimation methods of BF% and biomarkers

**Table 4:**
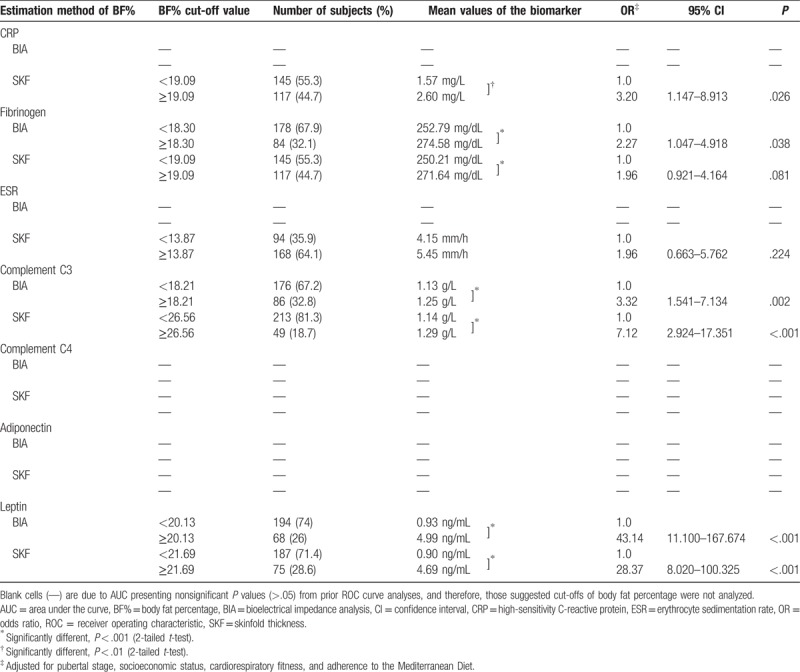
Number and percentage of boys below and above the cut-off value and respective mean values of the biomarker in question, and associations between the estimation methods of BF% and biomarkers

## Discussion

The main findings of this study suggest that both BIA and SKF were able to present discriminatory ability in identifying unfavorable levels of all biomarkers of cardiometabolic risk in girls, whereas in boys this was not verified for all the biomarkers. Furthermore, both estimation methods of BF% always showed higher AUCs for each biomarker in girls than in boys (with the only exception of BIA for leptin), suggesting that diagnostic performance is more accurate in girls. Although the differences between the AUCs of the 2 estimation methods of BF% for each biomarker were not statistically significant in an intra sex analysis, BIA always presented a trend for marginally superiors AUCs than SKF in girls (except for CRP), while in boys we did not verified a predominance of 1 method over the other, as we noted that SKF method showed higher AUCs for 3 biomarkers (CRP, fibrinogen, and ESR) and BIA for 2 of them (C3 and leptin). In addition, the estimated values of BF% in boys were significantly higher when assessed by SKF than by BIA, while in girls no significant differences between the BF% estimates provided by the 2 methods were observed.

As expected, for a similar BMI for age, girls and boys of this study were different in relation to its body composition. In line with the notion that in girls, unlike boys, body fat increases substantially during adolescence,^15,39^ in the present study girls presented, on average, more 9.53% of body fat than boys when assessed by BIA, and more 5.61% if assessed by SKF. Although BIA and SKF are estimating the same thing (BF%), and have shown strong partial correlations between them after adjustment for age and pubertal status (*r* = 0.82 for girls, *r* = 0.78 for boys, *P* < .001 for both, data not shown), the estimated values of BF% obtained by the 2 methods were not as similar for boys as they were for girls, as previously mentioned. While girls presented no significant differences between the mean values of BF% from the 2 methods (BIA: 25.39%; SKF: 25.15%, *P* = 0.374), in boys the mean values of BF% were significantly higher when assessed by SKF (19.54%) than with BIA (15.86%), *P* < .001.

Steinberger et al^40^ have shown that BF% calculated from the equations of Slaughter et al,^13^ using triceps and subscapular skinfolds, was strongly correlated with BF% determined by dual-energy X-ray absorptiometry (*r* = 0.93 for boys and 0.92 for girls, *P* < .0001). Nevertheless, in another study^41^ with Spanish adolescents, it was considered that the same equations might have overestimated BF% in males and underestimated BF% in females, when compared with dual-energy X-ray absorptiometry assessments.

Other studies provided results indicating differences in estimates of BF% obtained by BIA or by SKF in children and adolescents.^42^ Sexual dimorphism in the distribution of the body fat between boys and girls^43^ could be a possible explanation for the differences between the values obtained by the 2 methods in boys from our study. Given that boys generally have more lean mass in the lower limbs and more fat mass in the upper body, while girls usually present a more uniform and peripheral distribution of subcutaneous fat,^44^ the lower values of BF% assessed by BIA (through a foot-to-foot device), and the higher values when assessed by SKF (with trunk and arm measurements but no lower limbs information), may be reflecting these regional differences in body fat distribution in boys. That is, BIA may be showing some difficulties in estimating fat mass where it can be scarce, underestimating it, while SKF may indeed be overestimating the amount of fat mass due to not consider measurements in lower limbs. However, the fact that no gold standard method was used in this study prevents us to infer about the possible underestimation or overestimation of BF% by each method, since we cannot confirm what is the “true” BF% of each adolescent.^45^

Nevertheless, whether measured by BIA or SKF, our results support the idea that adiposity is a relevant determinant of a diversity of biomarkers of cardiometabolic risk during adolescence,^5,6^ just like it is in children.^46^ The higher percentage of body fat in girls seems to make the relationships between adiposity and biomarkers more evident than in boys, with girls presenting higher levels in most of them, with the exception of CRP. Although the levels of this inflammatory marker are usually higher in females, results similar to ours were also reported in other studies with adolescents.^47–49^

Despite no significant differences between the AUC of each measure for any given biomarker were found, and in the case of girls, BIA and SKF have provided identical mean values of BF% (25.39% vs 25.15%, respectively), the ROC curves analyses suggested different cut-points of BF% if measured by BIA or by SKF, to identify increased values of biomarkers, suggesting that the 2 methods are not interchangeable for this purpose.

The range of the cut-off values of BF% assessed by BIA, suggested by valid ROC curves analyses (ie, AUC > 0.5 and *P* < .05) to predict increased levels of several biomarkers, varies from 20.32% (for ESR) to 30.69% (for C3) in girls, and from 18.21% (for C3) to 20.13% (for leptin) in boys. The top values of these intervals are very close to the age- and sex-specific 85th percentiles of BF% (measured by BIA) indicatives of overfat for each sex, suggested by McCarthy et al.^15^ Accordingly, adolescents are considered overfat at the age of 14 if they have more than 29.6% and 21.3% of body fat, and at the age of 15 if they have more than 29.9% and 20.7%, for girls and boys, respectively (this age interval of 14–15 years old corresponds to the mean age of the sample of this study). Nevertheless, all the cut-offs of BF% suggested by valid AUC identifying unfavorable levels of some biomarkers in boys (fibrinogen, 18.30%; C3, 18.21%; leptin, 20.13%) were below the aforementioned 85th percentile of McCarthy defining overfat for this age, and for girls, only 2 biomarkers (C3 and adiponectin) were above the same percentile.

For example for CRP, and using the cut-off value suggested by the ROC analysis of BF% when obtained by BIA, our results indicate that girls presenting a BF% value ≥24.4%, have a mean value of CRP of 1.11 mg/L (placing them in the intermediate risk adult cut-off^21^), and are 8.41 times more likely to have significantly higher levels of CRP, when compared with the girls below that cut-off of BF%, who presented an average value of 0.39 mg/L of CRP (Table 3). The same interpretation should be made when analyzing data from measurements with SKF for the same biomarker, that is, girls with values of BF% equal to or above the cut-off of 23.93% present a mean value of CRP of 1.22 mg/L, and are 13.16 times more likely to have higher levels than the girls below the cut-off, which present a mean value of CRP of only 0.37 mg/L.

These results suggest that, even at cut-off values of BF% below of what corresponds to the 85th percentile threshold identifying overfat by McCarthy et al,^15^ adolescents could present an indicative trend for developing an unfavorable profile for some biomarkers. The detailed analysis of the data presented in Tables 3 and 4 allows us to distinguish 2 different subgroups presenting significant different values on the concentrations of several biomarkers, depending on whether subjects are above or below the cut-off suggested by the ROC curves analyses for identify an unfavorable level of the respective biomarker.

Some limitations of this study should be mentioned. First, its cross-sectional design does not allow us to assess the directionality of the relationships between both methods of BF% estimation and the different biomarkers; second, the use of a leg-to-leg BIA instead of a tetrapolar BIA may be a limitation in this study, due to the typical fat distribution in adolescents; third, a criterion method to compare with the 2 analyzed methods of BF% estimation was not used, thus, making the inferences about under or overestimation of BF% values by each method uncertain, so, future research should consider the inclusion of a gold standard test, in order to clarify this point. Although the method validated by Tanner and colleagues is widely used in studies of epidemiological characteristics involving children, because of the greater logistical ease in large samples, and its noninvasive nature, this method may not be as accurate as the direct observation of children by a pediatrician. Lastly, the use of a unique measure of each biomarker may not accurately represent a long-term pattern of that specific biomarker.

Nevertheless, in an attempt to minimize and improve this last point, we analyzed not one, but several biomarkers, which provide us a more consistent scenery of the cardiometabolic status. Another strength of this study is that our results indicate that 2 simple and largely used methods of BF% estimation, may be seen as an indirect strategy that could provide an overview of several inflammatory and metabolic biomarkers of a sample of adolescents. This hypothesis may be of special interest in epidemiological settings, when a large number of subjects are assessed and, due to financial, time or legal constraints, blood samples cannot be easily taken.

In conclusion, the main findings of this study suggest that both methods present discriminatory ability in identifying unfavorable levels in all biomarkers of cardiometabolic risk in girls, whereas in boys this was not observed. Moreover, in girls, BIA presented a slightly better overall diagnostic performance for each biomarker than SKF, while in boys no method clearly prevailed over the other.

## Acknowledgments

This work was supported by the Portuguese Foundation for Science and Technology (J.O.-S., grant number SFRH/BD/88984/2012), (R.S., grant number SFRH/BPD/102381/2014), and CIAFEL is supported by UID/DTP/00617/2013; R.S. has a Discovery Early Career Research Award number DE150101921 from the Australian Research Council; C.A.-S. is supported by the Brazilian Government by the Coordination of Improvement of Higher Education Personnel—CAPES (grant number 9588-13-2). The Portuguese Foundation for Science and Technology, the Australian Research Council, and the Brazilian Government by CAPES had no role in the design, analysis, or writing of this article.

## Conflicts of interest

The authors declare no conflicts of interest.

## Abbreviations

Abbreviations: AUC = area under the curve, BF% = body fat percentage, BIA = bioelectrical impedance analysis, C3 = complement factor C3, C4 = complement factor C4, CRP = high-sensitivity C-reactive protein, ESR = erythrocyte sedimentation rate, FAS = Family Affluence Scale, ROC = receiver operating characteristic, SKF = skinfold thickness, VO_2max_ = maximal oxygen consumption.
